# The Delayed Neuropathological Consequences of Traumatic Brain Injury in a Community-Based Sample

**DOI:** 10.3389/fneur.2021.624696

**Published:** 2021-03-16

**Authors:** Nadia Postupna, Shannon E. Rose, Laura E. Gibbons, Natalie M. Coleman, Leanne L. Hellstern, Kayla Ritchie, Angela M. Wilson, Eiron Cudaback, Xianwu Li, Erica J. Melief, Allison E. Beller, Jeremy A. Miller, Amber L. Nolan, Desiree A. Marshall, Rod Walker, Thomas J. Montine, Eric B. Larson, Paul K. Crane, Richard G. Ellenbogen, Edward S. Lein, Kristen Dams-O'Connor, C. Dirk Keene

**Affiliations:** ^1^Department of Laboratory Medicine and Pathology, University of Washington School of Medicine, Seattle, WA, United States; ^2^Department of Medicine, University of Washington School of Medicine, Seattle, WA, United States; ^3^Allen Institute for Brain Science, Seattle, WA, United States; ^4^Kaiser Permanente Washington Health Research Institute, Seattle, WA, United States; ^5^Department of Pathology, Stanford University School of Medicine, Palo Alto, CA, United States; ^6^Department of Neurological Surgery, University of Washington School of Medicine, Seattle, WA, United States; ^7^Department of Neurology, Icahn School of Medicine at Mount Sinai, New York, NY, United States

**Keywords:** chronic traumatic encephalopathy, histelide, Adult Changes in Thought study, amyloid–beta, traumatic brain injury, immunohistochemistry, hyperphosphorylated tau

## Abstract

The late neuropathological effects of traumatic brain injury have yet to be fully elucidated, particularly with respect to community-based cohorts. To contribute to this critical gap in knowledge, we designed a multimodal neuropathological study, integrating traditional and quantitative approaches to detect pathologic changes in 532 consecutive brain autopsies from participants in the Adult Changes in Thought (ACT) study. Diagnostic evaluation including assessment for chronic traumatic encephalopathy (CTE) and quantitative immunoassay-based methods were deployed to examine levels of pathological (hyperphosphorylated) tau (pTau) and amyloid (A) β in brains from ACT participants with (*n* = 107) and without (*n* = 425) history of remote TBI with loss of consciousness (w/LOC). Further neuropathological assessments included immunohistochemistry for α-synuclein and phospho-TDP-43 pathology and astro- (GFAP) and micro- (Iba1) gliosis, mass spectrometry analysis of free radical injury, and gene expression evaluation (RNA sequencing) in a smaller sub-cohort of matched samples (49 cases with TBI and 49 non-exposed matched controls). Out of 532 cases, only 3 (0.6%–none with TBI w/LOC history) showed evidence of the neuropathologic signature of chronic traumatic encephalopathy (CTE). Across the entire cohort, the levels of pTau and Aβ showed expected differences for brain region (higher levels in temporal cortex), neuropathological diagnosis (higher in participants with Alzheimer's disease), and *APOE* genotype (higher in participants with one or more *APOE* ε4 allele). However, no differences in PHF-tau or Aβ_1−42_ were identified by Histelide with respect to the history of TBI w/LOC. In a subset of TBI cases with more carefully matched control samples and more extensive analysis, those with TBI w/LOC history had higher levels of hippocampal pTau but no significant differences in Aβ, α-synuclein, pTDP-43, GFAP, Iba1, or free radical injury. RNA-sequencing also did not reveal significant gene expression associated with any measure of TBI exposure. Combined, these findings suggest long term neuropathological changes associated with TBI w/LOC may be subtle, involve non-traditional pathways of neurotoxicity and neurodegeneration, and/or differ from those in autopsy cohorts specifically selected for neurotrauma exposure.

## Introduction

Traumatic brain injury (TBI), defined as a blow or jolt to the head or neck that results in altered brain function, is a major cause of morbidity and mortality and a public health crisis. It is estimated that TBI affects nearly three million people in the US per year, not including undiagnosed and untreated cases (1). Furthermore, at least 2% of the US population is living with chronic disability related to a prior head trauma (2, 3). Population-based studies suggest that this prevalence may be substantially higher (4).

The consequences of TBI are not necessarily limited to the injury sustained in the acute stage and frequently lead to chronic dysfunction, even in mild TBI (5). Progressive degeneration of the brain on imaging with time has also been identified in some cases (6). However, the late neuropathological features associated with TBI are not yet well-characterized. While many cases of significant exposure to repetitive head trauma (e.g., extended exposure to contact sports, military service, or domestic violence) are associated with chronic traumatic encephalopathy (CTE), with sulcal and perivascular accumulation of phosphorylated tau (pTau) in neurons and glia (7–10), there is less understanding of the effects of the most common type of TBI in the general population- single TBI or multiple isolated TBI(s) that are associated with altered mental status and/or loss of consciousness (LOC). These injuries are much more common than the prolonged exposure to repetitive concussive/subconcussive head trauma experienced in high level contact sport associated with the CTE neuropathologic change (7, 11, 12), and often result from common life activities, such as motor vehicle accidents or falls.

A growing interest in the late neuropathological/neurodegenerative impact of TBI is fueled by studies that link TBI with later development of dementia, the risk appearing to increase with the number and severity of TBIs (13–18). Lee et al. (19) reported that even a single mild TBI may increase the lifelong risk for dementia; a history of TBI also appears to lower the age of dementia onset (18, 20). Research supports a link between moderate and severe TBI and development of Alzheimer's disease (AD) dementia (13, 14, 21, 22). TBI exposure has also been associated with frontotemporal lobar degeneratin (FTLD) (23–25), amyotrophic lateral sclerosis (ALS) (26), and Parkinson's disease (PD), where early life TBI exposure was more strongly predictive of PD and Lewy body disease (LBD) (27). However, many well-controlled studies have not found any effect of prior TBI on risk of dementia or AD (25, 27–33).

To date, the pathology thought to characterize late structural impact of repetitive concussive/subconcussive neurotrauma is CTE (7, 34–36). The specific mechanism(s) linking this pattern of neurotrauma with the later sulcal and perivascular pTau deposition, the pathognomonic feature of CTE (9) has not been fully elucidated, but pathological forms of tau have been observed in brain tissue and CSF post-injury (34, 37, 38). Whether CTE neuropathology is unique to repetitive subconcussive/concussive head trama experienced by high level contact sport athletes, military service members, or those exposed to intimate partner violence, continues to be investigated (3). The pTau pathology of CTE and/or a history of TBI is also often associated with other pathologic protein depositions. Amyloid (A)β plaques have been detected in proximity to CTE-specific lesions (7, 8, 39). Accumulation of TAR DNA-binding protein 43 (TDP-43), a nuclear protein implicated in the development of FTLD-TDP and ALS, has also been described in cases of repetitive brain trauma, with or without CTE diagnosis in humans (8, 25, 40–42), as well as animal models (43). α-Synuclein, a synaptic protein characteristically present in Lewy bodies (LBs) and required for pathologic diagnosis of PD/Lewy body dementia, has also been found in the brains of patients with a history of remote TBI (27, 44), in CSF after acute TBI (45, 46), and in animal models of brain trauma (47). Persistent inflammation may also play an important role in the development of post-TBI pathology. Several studies have shown that the inflammatory process chronically persists in TBI patients following trauma (48–52).

Given the high prevalence of TBI with associated LOC in the general population, and the widespread participation in recreational activity and contact sports among children and young adults, it is essential to develop a better understanding of the long-term neuropathological effects of non-repetitive TBI. To address this important knowledge gap, we investigated the delayed neuropathological consequences of TBI in a community-based sample. We not only screened for the presence of CTE pathology in neocortical sections, but we also measured the levels of pathological proteins (including several forms of pathological tau, Aβ, α-synuclein, and TDP-43) with multiple modalities including immunohistochemistry in formalin-fixed paraffin-embedded (FFPE) and flash-frozen tissue, a multiplexed Luminex assay in flash frozen tissue, and Histelide in FFPE. The inflammatory response was also assessed by evaluating astrogliosis and microgliosis, and free radical injury/oxidative stress by measuring isoprostane species. Finally, bulk RNA-sequencing was evaluated to screen for a potential genetic signature of remote TBI.

## Materials and Methods

### Study Design and Experimental Cohorts

This study was approved by the University of Washington and Kaiser Permanente Research Institute Institutional Review Boards. All subjects were participants in the Adult Changes in Thought (ACT) study, described in detail elsewhere (53). Briefly, ACT study participants are community-dwelling members of the Kaiser Permanente health maintenance organization, aged 65 and older and without a dementia diagnosis at the time of enrollment. Information about TBI was obtained solely via self-report upon enrollment in the study using the single-item question, “Have you ever sustained a head injury so severe it resulted in loss of consciousness?” and at every study visit using the question “Since your last study visit, have you had a head injury so severe it resulted in loss of consciousness?” If a head injury w/LOC was reported, participants were then queried to determine the duration of LOC, age at injury, and whether medical attention was sought. Duration of LOC was classified as: a few seconds or less, a minute or less, 1–2 min, 3–5 min, 6–9 min, 10 minto 1 h, more than 1 h. Information about sport and military exposure was obtained from obituaries, which were available for 52% of cases. Cognitive status was assessed biannually by the Cognitive Abilities Screening Instrument (CASI) (54), which ranges from 0 to 100 and higher scores indicate better cognitive functioning. A CASI score of <86 indicated possible cognitive impairment and initiated a standardized dementia diagnostic evaluation, including a comprehensive neuropsychological battery and a neurological evaluation. All of these data were reviewed in a multidisciplinary consensus conference. Dementia diagnoses were assigned by consensus using *DSM-IV* criteria (55). All neuropathologic evaluations were performed according to current consensus protocols and methods at the time of autopsy. Apolipoprotein E (*APOE*; M12529) genotype was determined according to published methods (56).

To study the late neuropathological effects of TBI in ACT, we selected 532 consecutive ACT participant brain autopsies (TBI_All_ cohort), starting with the first ACT brain donor, from which subjects were divided into 2 groups by history of TBI with LOC: TBI group with 1-3 instances of TBI with LOC, duration ranging from a few seconds to more than an hour, *n* = 107, and an unexposed group with no history of TBI with LOC, *n* = 425. See Table 1 for cohort demographics.

**Table 1:** Study cohorts.

	**TBI_All_ cohort**		**TBI_1h/Multi_ ohort**		**TBI_Froz_ cohort**	
	**TBI**	**Control**	**LOC over 1 hour or multiple TBI-LOC**	**Control**	**TBI**	**Control**
Total (#)	107	425	27	53	49	52
TBI with LOC (# per case)	1.2 ± 0.5	0	1.8 ± 0.6	0	1.2 ± 0.5	0
LOC duration						
< min	28%		12%		38%	
1–9 min	42%		16%		24%	
10–60 min	14%		16%		19%	
Over 60 min	15%	N/A	56%	N/A	19%	N/A
Age at first LOC						
Under 25	55%		70%		53%	
25–64	15%		11%		14%	
65 and over	30%	N/A	19%	N/A	33%	N/A
Men	64%	42%	78%	77%	63%	62%
Age at death (years)	86.9 ± 6.9	87.9 ± 6.6	87.9 ± 6.4	88.1 ± 5.9	89.0 ± 6.4	89.1 ± 6.5
Dementia	45%	43%	52%	49%	49%	46%
Age at dementia diagnosis (years)	83.9 ± 6.5	84.7 ± 5.6	86.3 ± 5.8	85.3 ± 5.4	84.6 ± 6.0	85.8 ± 5.9
CASI score at enrollment	93.1 ± 3.8	93.3 ± 4.2	92.5 ± 3.0	93.8 ± 3.4	93.2 ± 3.2	93.0 ± 3.9
Last CASI score	83.8 ± 13.3	82.7 ± 15.9	84.7 ± 12.4	81.1 ± 16.9	84.8 ± 12.4	82.3 ± 16.0
PD diagnosis	0.9%	1.6%	0%	0%	0%	2.0%
ApoE ε4 allele carriers	22%	30%	11%	31%	18%	23%
Braak stage (median)	III	III	III	III	IV	III
CERAD score (median)	Sparse	Moderate	Sparse	Sparse	Sparse	Sparse
Micro VBI (microinfarcts ≥2)	33%	34%	37%	45%	27%	38%
Lewy bodies (any region)	15%	14%	15%	15%	17%	13%
Lewy bodies (neocortex)	5.7%	4.3%	7.4%	2%	6.3%	7.7%

*Data is represented as mean ± SD or percent. Micro VBI, microvascular brain injury; LBD, Lewy body disease; PD, Parkinson's disease; CASI, Cognitive Abilities Screening Instrument*.

Samples of FFPE brain tissue were obtained from middle frontal gyrus (MFG, Brodmann area nine), superior and middle temporal gyrus (SMTG), and inferior parietal lobule (IPL). Levels of pTau and Aβ_1−42_ were quantified in all three regions using the Histelide immunoassay (57). MFG, SMTG, and IPL sections immunolabeled for pTau were also screened for signs of CTE.

**Figure 1:**
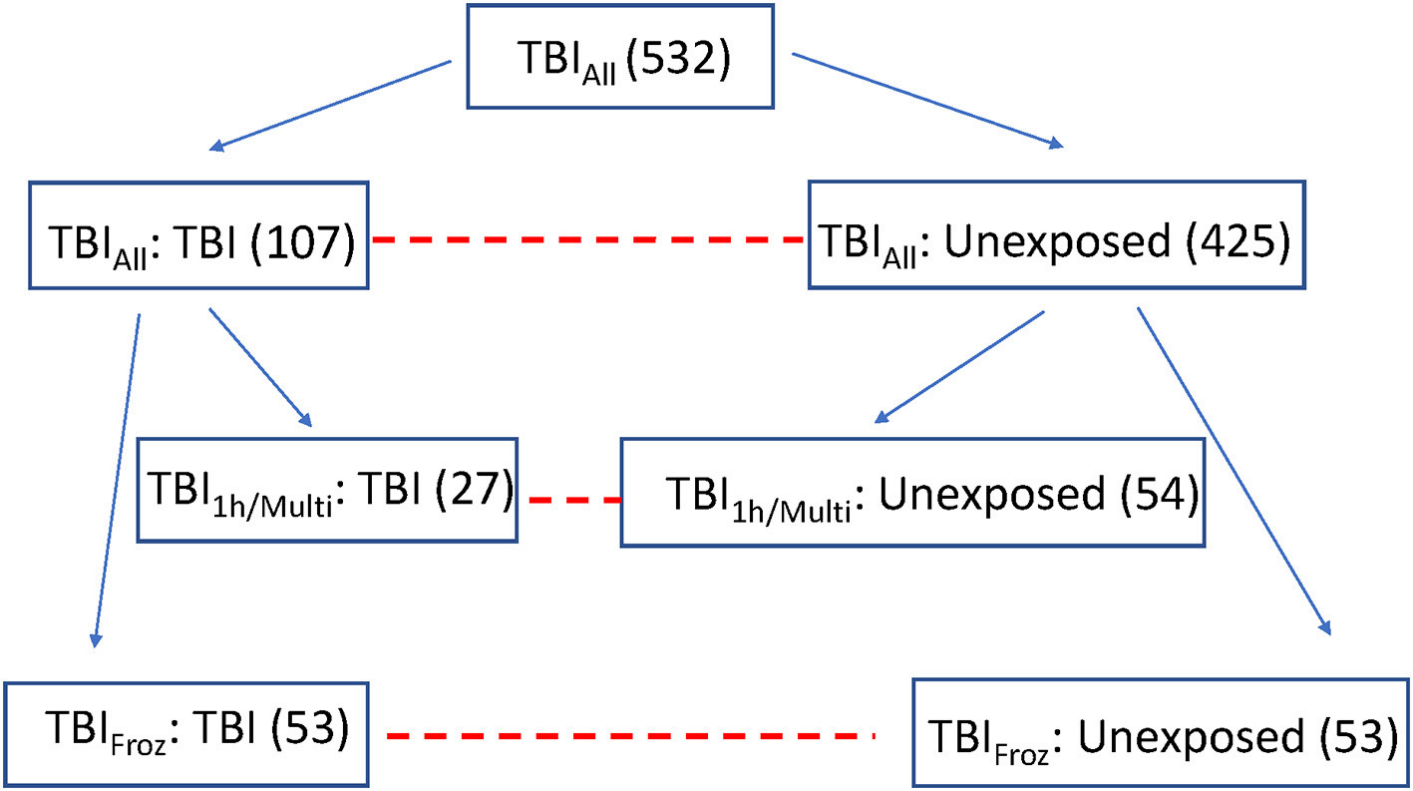
Overview of the experimental cohorts. Dashed lines connect comparison groups.

To focus in more depth on people with the highest TBI exposure, we used the original TBI cohort (TBI_All_) to identify participants with the most significant TBI exposure, defined as a single TBI with LOC >1 h and/or >1 TBI with LOC (TBI_1h/Multi_, *n* = 27). Each of the 27 TBI_1h/Multi_ participants was matched with two unexposed cases from the original control cohort, with matching by sex, age, year of death, and post-mortem interval (see Figure 1). We used the Histelide immunoassay to quantify pTau in an expanded set of brain regions in these individuals, including the contralateral MFG, SMTG and IPL (data from both sides were averaged), as well as occipital cortex (calcarine fissure), anterior cingulate cortex, and hippocampus with adjacent entorhinal cortex at the coronal level of lateral geniculate nucleus). See Table 2 for more information about the regions examined and analyses.

**Table 2:** Overview of experimental methods and analytes in respect to the experimental cohorts.

**Method (region)**	**TBI_**All**_**	**TBI_**1h/Multi**_**	**TBI_**Froz**_**
Histelide (MFG, SMTG, IPL)	pTau (AT8), Aβ_1−42_	pTau (AT8), Aβ_1−42_	pTau (AT8), Aβ_1−42_
Histelide (contralateral MFG, SMTG, IPL, occipital, cingulate, hippocampus)		pTau (AT8)	
IHC in FFPE tissue (SMTG, IPL, PWM, hippocampus)			pTau (AT8), pTau (Tau-2), Aβ (6E10), α-synuclein (LB509), pTDP-43 (1D3), GFAP, Iba1
IHC in flash-frozen tissue (SMTG, IPL, PWM, hippocampus)			pTau (AT8), Aβ (6E10), α-synuclein (LB509)
Luminex assay (SMTG, IPL, PWM, hippocampus)			pTau (p181), Aβ_1−42_, α-synuclein
Isoprostane measurements (SMTG, IPL)			Isoprostanes
RNA-Sequencing (SMTG, IPL, PWM, hippocampus)			RNA-Sequencing

To study the effect of TBI on brain using methods not optimized for formalin fixation, such as RNA-sequencing, measurements of protein concentrations, and oxidative stress, we identified 53 ACT participant autopsies with TBI w/LOC history (as defined above) and fresh frozen samples derived at rapid autopsy (fresh dissection of the brain with samples flash frozen in liquid nitrogen occurs in donors with post-mortem interval < 8 h and is referred to as a rapid autopsy). Matched controls (53) with flash frozen tissue and no history of TBI w/LOC were selected using the same control matching algorithm as above (TBI_Froz_ cohort). See Figure 1, Tables 1, 2 for more information. All experiments and analyses in the TBI_Froz_ cohort were carried out in collaboration with the Allen Institute for Brain Science (www.alleninstitute.org) and all data are freely available at http://aging.brain-map.org.

We studied samples of FFPE brain tissue and tissue flash-frozen at the time of autopsy from SMTG, IPL, and hippocampus. Parietal white matter (PWM) samples were obtained from IPL blocks. Cortical and hippocampal frozen samples, and cortical FFPE tissue blocks were taken from the same side of the brain within cases; sidedness for frozen sampling was randomly chosen at autopsy. FFPE hippocampal samples were taken from the opposite side, as rapid protocols dictated complete sampling of rapid side hippocampus for freezing. To allow for measurement of protein concentrations and oxidative stress, and visualization of pathology by immunohistochemistry (IHC) to be carried out in the same tissue samples, two adjacent blocks of frozen SMTG, IPL, and hippocampal tissue were used, with one divided for isoprostane and Luminex studies, and the other for frozen section immunohistochemistry and RNA-sequencing. If only one block was available, the cortical blocks were sufficiently large (~1.5 g) to be divided and used for each assay, but hippocampal blocks were small and therefore used only for immunohistochemistry/RNA-sequencing and not available for protein/isoprostane measurements (11 cases). Punch biopsies (1 cm) were used to selectively collect PWM, SMTG and IPL gray matter. In all cases, punches of cerebral cortex were targeted to the depth of sulcus region and white matter punches were targeted to the deepest available layer in each tissue block. The hippocampus was sampled in its entirety; http://aging.brain-map.org for additional details.

### Histelide in FFPE Tissue

Histelide immunoassays were carried out as previously published (57). Briefly, 5 μm sections of FFPE tissue were placed on charged microscope slides. Slides were deparaffinized, rehydrated and subjected to antigen retrieval. Slides were incubated in normal goat serum/bovine serum albumin (NGS/BSA) blocking solution, anti-pTau or anti-amyloid (A)β_1−42_ primary antibody (see Table 3), and alkaline phosphatase-conjugated goat secondary antibody (Jackson ImmunoResearch Laboratories, West Grove, PA, USA). Slides were then incubated in p-nitrophenyl phosphate (PNPP) solution, and the soluble chromogenic PNP product absorbance was read at 405 nm by a spectrophotometer (Molecular Devices SpectraMax). To allow for visualization of antibody deposits, the slides were then placed into insoluble chromogen nitro-blue tetrazolium/5-Bromo-4-chloro-3-indolyl phosphate (NBT/BCIP, Sigma-Aldrich, St. Louis, MO, USA) and coverslipped. Gray and white matter areas were traced using a Nikon 90 microscope and Stereo Investigator software (MBF Bioscience, Williston, VT). Background signal was calculated using negative control slides included in each run. PNPP signal minus background was normalized to gray matter area to give the signal intensity of gray matter (SI_GM_, Abs/cm^2^).

**Table 3:** Antibodies and pretreatments.

**Primary antibody**	**Clone**	**Manufacturer (Product number)**	**Assay**	**Pretreatments**	**Antibody dilution**
pTau	AT8	Pierce (MN10120)	Histelide	Formic acid	1:500
			IHC in FFPE tissue	ER2 (30 min)	1:2,000
			IHC in frozen tissue	Acetone Fix, Sodium Citrate	1:1,000
Amyloid β_1−42_	H31L21	Invitrogen (700254)	Histelide	Formic acid	1:1,000
Amyloid β	6E10	Biolegend (SIG-39320)	IHC in FFPE tissue	ER2 (20 min)	1:2,000
			IHC in frozen tissue	Acetone Fix, Sodium Citrate	1:2,000
	Tau-2	Sigma-Aldrich (T5530)	IHC in FFPE tissue	ER2 (10 min)	1:5,000
α-synuclein	LB509	Life Technologies (18-0215)	IHC in FFPE tissue	ER2 (20 min)	1:2,000
			IHC in frozen tissue	Acetone Fix, Sodium Citrate	1:2,000
Phospho-TDP-43	1D3	Millipore (MABN14)	IHC in FFPE tissue	ER2 (20 min)	1:250
GFAP	N/A	Dako (Z0334)	IHC in FFPE tissue	ER1 (20 min)	1:10,000
Iba-1	N/A	Wako (019-19741)	IHC in FFPE tissue	ER2 (20 min)	1:1,700

### Histological Staining and IHC in FFPE Tissue

FFPE tissue blocks were cut at 5 μm thickness and processed using standard histological techniques. Sections of SMTG, IPL and hippocampus were stained with hematoxylin and eosin/Luxol Fast Blue (H&E/LFB) and immunolabeled for Aβ (clone 6E10), pTau (clone AT8), phosphorylated and non-phosphorylated pathologically modified tau (Tau-2), phospho-TDP-43 (1D3), GFAP, and Iba1 (see Table 3). More detailed descriptions of the methods are available at http://aging.brain-map.org.

### Histological Staining and IHC in Flash-Frozen Tissue

Tissue blocks were cut at 25 μm thickness, processed using standard histological techniques and stained with cresyl violet for anatomical reference. The sections of SMTG, IPL and hippocampus were also immunolabeled using antibodies to Aβ (clone 6E10), pTau (AT8), and α-synuclein (LB509). For more details, see Table 3 and http://aging.brain-map.org.

### Image Acquisition and Analysis

Histologically stained slides (frozen and FFPE) were scanned using the Leica ScanScope® automated slide scanner (Leica Biosystems). Images were acquired at 10x magnification. All images were first converted to JPEG 2,000 format, then oriented for consistency across slides and white balanced to achieve consistent white background intensities. Image analysis was carried out in a similar manner for frozen and FFPE tissue, except we applied a spectral filter to the FFPE tissue sections to attenuate the signal from the H&E/LFB stain. Expression density, defined as the percentage of the area within the region of interest (ROI) that was occupied by the IHC reaction product, was assessed algorithmically. To validate the metrics that were generated, data from antibodies used on both flash frozen and FFPE tissue was correlated. Good correlations were seen for both Aβ (*R* = 0.68, *p* = 6.5 × 10^−51^) and pTau (*R* = 0.78, *p* = 4.7 × 10^−76^). More detailed descriptions of all methods are available at http://aging.brain-map.org.

### Luminex Assay

Flash-frozen tissue samples from 1 cm punch biopsies of gray matter from the SMTG, IPL, hippocampus, and of white matter from the IPL (PWM) were subjected to sequential extractions and centrifugation, and concentrations of pathological proteins pTau phosphorylated at threonine 181 (p181), amyloid β_1−42_, and α-synuclein, were measured by multiplexed Luminex assays (Luminex Corporation, Austin, TX). The data was analyzed using the LiquiChip Workstation (Qiagen, Hilden, Germany).

### Measures of Oxidative Stress

Free radical injury was measured in the samples of flash-frozen SMTG and IPL using a stable isotope dilution assay with gas chromatography mass spectrometry (GC/MS) quantitation of isoprostanes according to the published protocol (58). GC/MS analysis was conducted using a 6890N Agilent gas chromatograph coupled to a 5,973-quadrupole mass spectrometer in the negative-ion mode. Areas under peaks for *m/z* 569.5 and 573.2 (internal standard) were manually integrated to quantify both analytes.

### RNA-Sequencing and Analysis in Flash-Frozen Tissue

Samples of MFG, SMTG, IPL, and PWM and hippocampus were collected by manual microdissection in tissue adjacent to that processed for IHC. Specific areas for macrodissection were identified by neuroanatomists using images of tissue sections labeled with cresyl violet and were excised from the remaining frozen tissue block using a scalpel. For a detailed protocol please see Miller et al. (59). We used surrogate variable analysis (SVA) (60) to quantify significance of gene expression after normalizing by read depth only, and after controlling for RNA quality (59). Next, we repeated our analyses on RIN-normalized data using additional statistical tests including (1) two tailed student *t*-tests, (2) ANOVA, (3) and limma (61). Finally, we used SVA to assess whether any of the top principal components or module eigengenes described in Miller et al. (59) showed significant association with TBI. Statistical analyses for assessing significance of RNA-sequencing data are described in Miller et al. (59). Code for reproducing this part of the analysis has been added to https://github.com/AllenInstitute/agedbrain.

### CTE Histology Screen

All sections of MFG, SMTG, and IPL immunolabeled with a pTau antibody as part of the Histelide assay were screened for an increase in tau at the depth of the sulcus. These cases were reviewed by a board-certified neuropathologist (A.L.N, C.D.K) and if an increase in tau accumulation in the deep gray matter at the sulcus was observed, additional evaluation of standard AT8 IHC stains was performed. These slides were evaluated for the pathognomonic lesion of CTE, according to consensus guidelines (9). The case was considered to have diagnostic neuropathologic evidence of CTE if any of the sections examined contained glial and neuronal pTau deposits at the depth of a sulcus in a clearly identified perivascular arrangement. Because of the limited number of regions analyzed, we did not assign a stage to any of the cases.

### Data Analysis

Poisson regressions with robust standard errors were used because the outcome measures were all highly skewed. A relative risk of two means, for example, that the average measurement is estimated to be twice as high in the group of interest (TBI group) compared to the reference group (unexposed group). We adjusted for age at death, sex, education, and the presence of 1 or more *APOE* ε4 alleles. Alpha was set at 0.05, 2-sided. All statistical analyses were carried out in Stata 16.1.

## Results

### Cohort Characteristics

In the TBI group of the TBI_All_ cohort, 68 cases (64%) were male, and the average age at death was 86.9 ± 6.9 years. Thirty-six (34%) participants were diagnosed with AD, and 11 (10%) were diagnosed with LBD or PD. In the unexposed group, 179 (42%) were male, and the average age at death was 87.9 ± 6.6 years. One hundred forty-six participants (34%) were diagnosed with AD, 44 (10%) were diagnosed with LBD or PD, 2 (0.5%) were diagnosed with PSP and 1 with FTLD. The incidence of all-cause dementia appeared higher in the TBI group (45%, or 46/107), compared to the unexposed group (43% or 183/425), but the difference was not statistically significant. Likewise, average CASI score at study enrollment, last CASI score before death and average age at dementia diagnosis were not significantly different between the two groups (see Table 1). Over half of the participants in the TBI group (59/107, or 55%) sustained their first injury before the age of 25, and in 30% (32/107) the first TBI occurred after 65 years of age. For more details about the TBI_All_ cohort, as well as TBI_1h/Multi_ and TBI_Froz_ cohorts, see Table 1.

### Histelide Measures of pTau and Aβ_1−42_ in TBI_All_ and TBI_1h/Multi_ Cohorts

**Figure 2:**
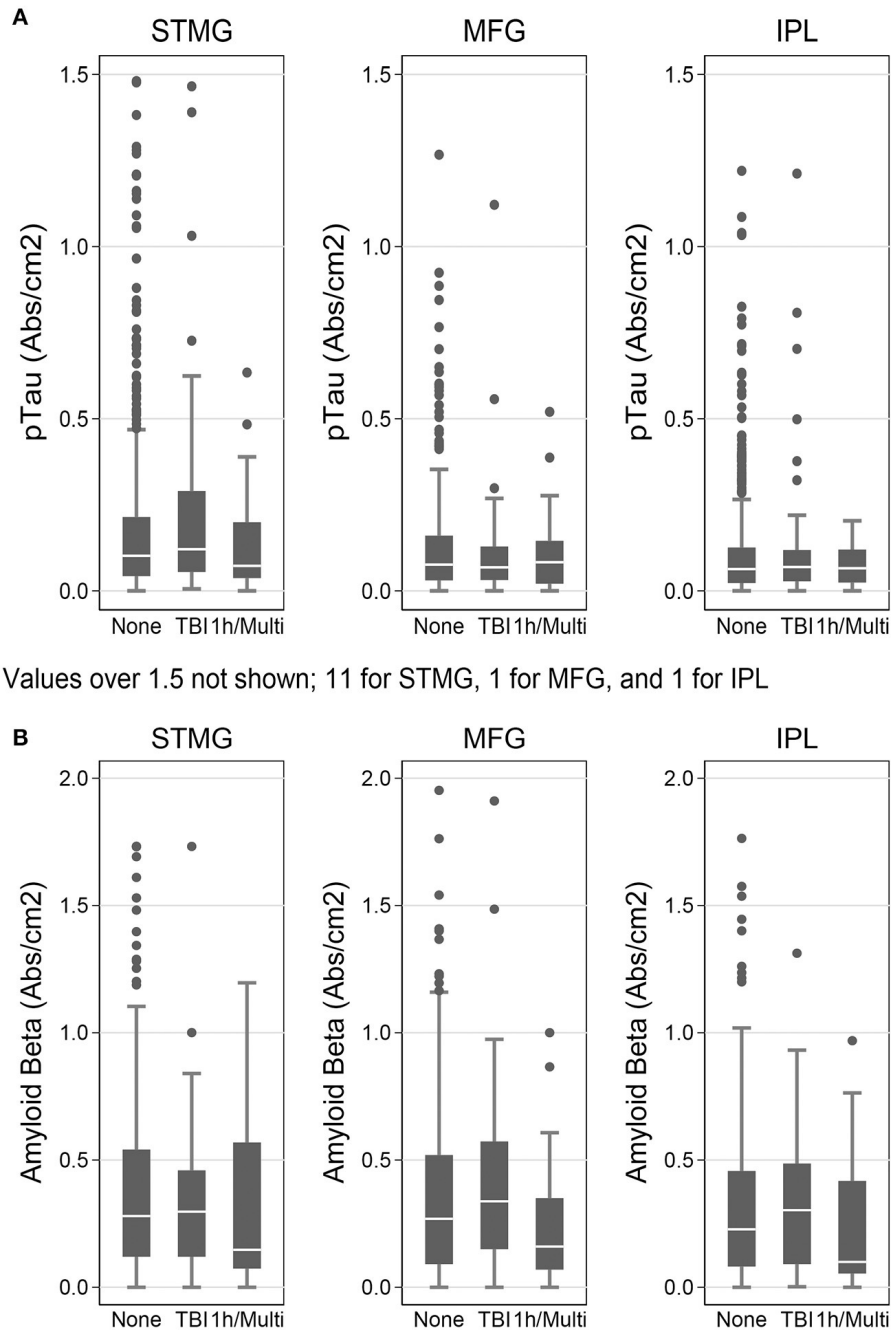
pTau and Aβ_1−42_ levels as measured by Histelide assay in the TBI_All_ cohort. pTau **(A)** and Aβ_1−42_ **(B)** were measured in the SMTG, MFG, and IPL. “TBI” indicates a history of a single TBI w/LOC of 1 h or less. “1h/Multi” indicates a history TBI w/LOC of more than 1 h or multiple TBIs. In the box-and-whiskers plots, the box spans the 25th to 75th percentiles, with the median indicated in white. The whiskers define 1½ times the inter-quartile range; individual observations more extreme than this are indicated with dots.

We used the Histelide method to measure pTau and Aβ_1−42_ levels in FFPE tissue samples from frontal, temporal and parietal cortex (MFG, SMTG, and IPL) in 532 ACT cases (TBI_All_ cohort): 107 cases with history of TBI exposure and 425 cases without history of TBI exposure. Data from a subset of these cases were reported earlier (57). There were no significant relationships between TBI exposure and any of the pTau or Aβ_1−42_ Histelide measures in MFG, SMTG or IPL, either for any TBI in the TBI_All_ sample (Figure 2, Table 4) or for those with LOC >1 h or multiple TBIs in the TBI_1h/Multi_ sample (Figure 2, Table 5).

**Table 4:** Relative Risks with 95% confidence intervals for any TBI with LOC in the TBI_All_ cohort.

**Analyte**	**Relative risk** **(95% CI)**	***p*-value**
*pTau (SI/cm^2^)*		
SMTG	0.99 (0.70, 1.40)	0.95
MFG	0.86 (0.64, 1.15)	0.31
IPL	0.86 (0.57, 1.31)	0.49
*Aβ_1−42_ SI/cm^2^)*		
SMTG	0.97 (0.81, 1.18)	0.79
MFG	1.12 (0.92, 1.36)	0.28
IPL	1.02 (0.85, 1.24)	0.81

*Levels of pTau and Aβ_1−42_ were assessed by Histelide. All models are adjusted for age at death, sex, education, and the presence of any APOE ε4 alleles*.

**Table 5:** Relative Risks with 95% confidence intervals for a single TBI-LOC lasting an hour or less and for LOC greater than an hour or multiple TBI-LOC in the TBI_All_ cohort.

	**Single TBI, LOC 1 h, or less**		**LOC over 1 h or multiple TBI**	
**Analyte *(SI/cm^**2**^)***	**Relative risk (95% CI)**	***p*-value**	**Relative risk (95% CI)**	***p*-value**
*pTau*				
SMTG	1.10 (0.75, 1.61)	0.63	0.65 (0.41, 1.03)	0.07
MFG	0.83 (0.57, 1.19)	0.31	0.95 (0.62, 1.46)	0.83
IPL	0.94 (0.60, 1.49)	0.80	0.63 (0.39, 1.02)	0.06
*Aβ_1−42_*				
SMTG	0.98 (0.79, 1.22)	0.86	0.95 (0.66, 1.38)	0.80
MFG	1.21 (0.98, 1.50)	0.08	0.84 (0.59, 1.17)	0.30
IPL	1.07 (0.87, 1.32)	0.52	0.88 (0.60, 1.28)	0.50

*All models are adjusted for age at death, sex, education, and the presence of any APOE ε4 alleles*.

*Levels of pTau and Aβ_1−42_ were assessed by Histelide*.

In the 27 people with LOC >1 h or multiple TBI (TBI_1h/Multi_) and in 53 matched individuals with no history of TBI, pTau levels were measured in bilateral MFG, SMTG or IPL and 3 additional regions- occipital cortex, cingulate cortex, and hippocampus (with adjacent entorhinal cortex included). Again, no significant differences were identified (Supplementary Table 1).

### PTau and Aβ_1−42_ Levels as Assessed by Immunohistochemistry and Luminex Assay in TBI_Froz_ Cohort

**Figure 3:**
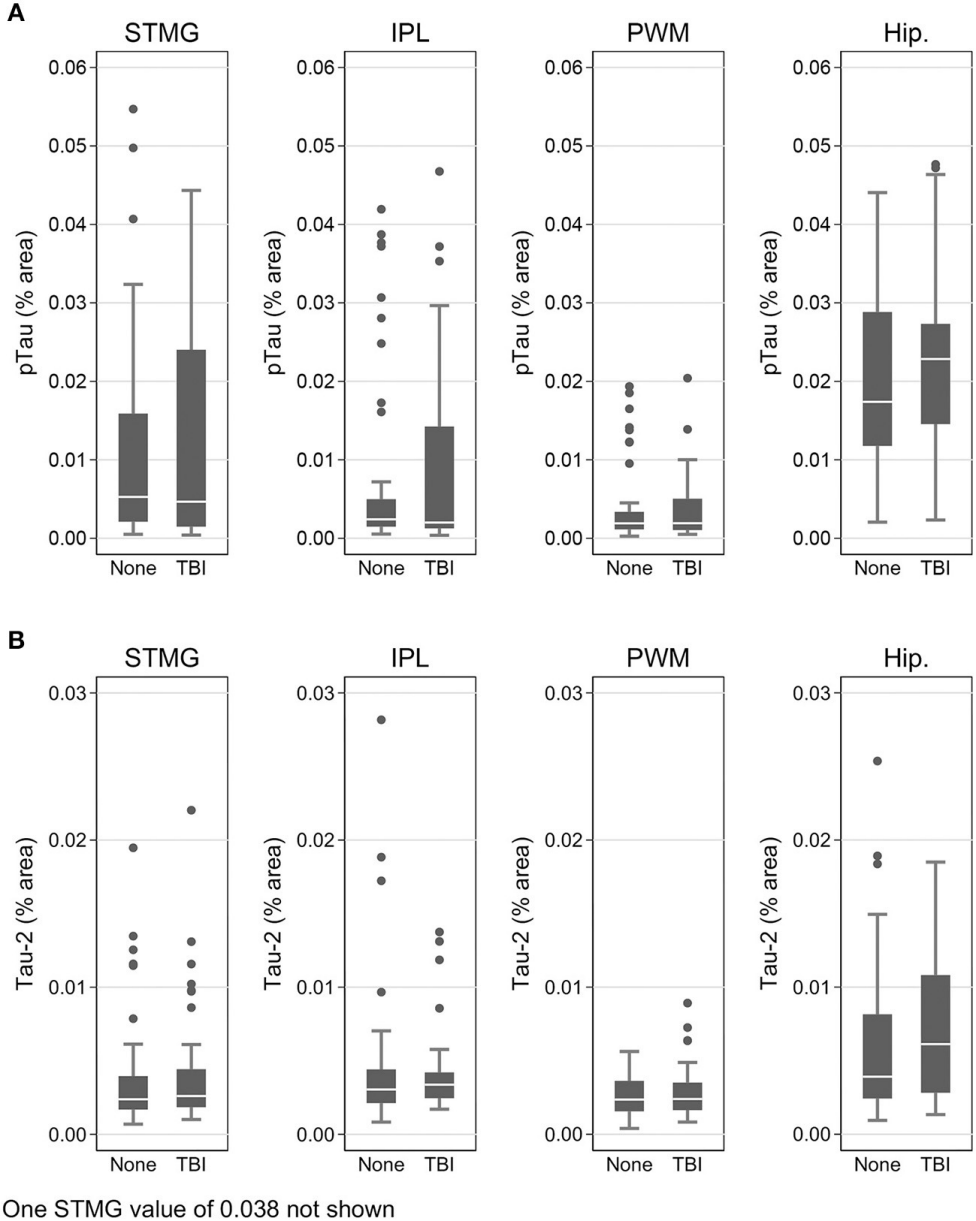
pTau levels in SMTG, IPL, hippocampus, and PWM in FFPE tissue samples in the TBI_Froz_ cohort. Pathological tau (% area occupied by stain) in TBI cases and matched controls as measured by IHC with AT8 antibody **(A)**, and Tau-2 antibody **(B)**. In the box-and-whiskers plots, the box spans the 25th to 75th percentiles, with the median indicated in white. The whiskers define 1½ times the inter-quartile range; individual observations more extreme than this are indicated with dots.

**Figure 4:**
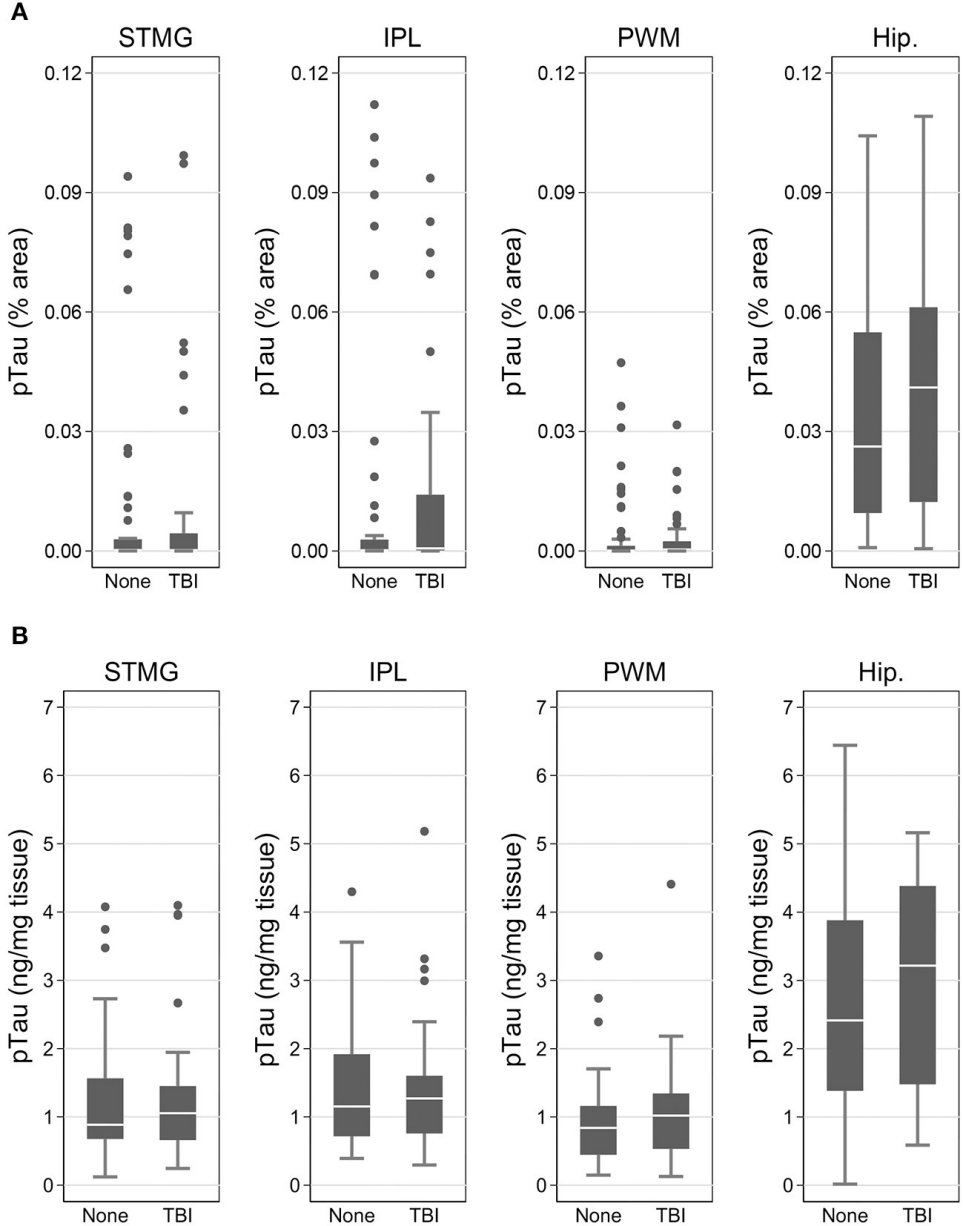
pTau levels in SMTG, IPL, hippocampus, and PWM in the in flash-frozen tissue samples TBI_Froz_ cohort. pTau (% area occupied by stain) in TBI cases and matched controls as measured by IHC in frozen tissue sections using the AT8 antibody **(A)** and Luminex assay **(B)**. In the box-and-whiskers plots, the box spans the 25th to 75th percentiles, with the median indicated in white. The whiskers define 1½ times the inter-quartile range; individual observations more extreme than this are indicated with dots.

Using immunohistochemistry, pTau signal (as a percentage of the tissue area occupied by the AT8 antibody immunostain) was measured in FFPE and frozen tissue samples and assessed with Tau-2 antibody in FFPE samples alone, from SMTG, IPL, hippocampus and PWM. As shown in Figures 3, 4A, Table 6, the only differences observed between people with a history of TBI w/LOC and people with no history of TBI were higher levels of pTau as assessed with AT8 and Tau-2 antibodies in FFPE tissue in hippocampus, as indicated by the higher risk ratios. PTau signal measured in frozen tissue samples by Luminex assay was not different between the two groups in any of the regions examined (Figure 4B).

**Table 6:** Relative Risks with 95% confidence intervals TBI compared to no TBI, in the TBI_Froz_ cohort.

	**SMTG**	**IPL**	**PWM**	**Hippocampus**
**IHC-FFPE (% area)**				
pTau	1.29 (0.85, 1.95), *p* = 0.24	1.55 (0.88, 2.73), *p* = 0.13	1.14 (0.73, 1.75), *p* = 0.57	1.29 (1.05, 1.60), ***p*** **= 0.02**
Tau-2	1.09 (0.73, 1.61), *p* = 0.68	1.05 (0.78, 1.42), *p* = 0.74	1.15 (0.90, 1.47), *p* = 0.26	1.37 (1.03, 1.82), ***p*** **= 0.03**
Aβ	0.84 (0.64, 1.10), *p* = 0.20	0.83 (0.63, 1.09), *p* = 0.19	1.03 (0.78, 1.34), *p* = 0.85	1.03 (0.83, 1.29), *p* = 0.77
pTDP-43	1.09 (0.90, 1.31), *p* = 0.39	1.01 (0.86, 1.18), *p* = 0.93	0.99 (0.83, 1.19), *p* = 0.91	1.07 (0.87, 1.31), *p* = 0.53
GFAP	1.03 (0.86, 1.23), *p* = 0.78	0.98 (0.84, 1.14), *p* = 0.78	1.01 (0.85, 1.19), *p* = 0.92	0.93 (0.80, 1.09), *p* = 0.37
Iba1	0.94 (0.83, 1.07), *p* = 0.35	0.93 (0.80, 1.08), *p* = 0.34	0.90 (0.78, 1.04), *p* = 0.15	1.00 (0.85, 1.16), *p* = 0.95
**IHC-Flash-frozen (% area)**				
pTau	1.16 (0.46, 2.92), *p* = 0.75	1.51 (0.68, 3.34), *p* = 0.31	0.84 (0.36, 1.98), *p* = 0.69	1.32 (0.96, 1.80), *p* = 0.09
Aβ	1.19 (0.83, 1.72), *p* = 0.35	1.13 (0.79, 1.63), *p* = 0.50	1.07 (0.66, 1.73), *p* = 0.78	1.01 (0.79, 1.30), *p* = 0.91
α-synuclein	0.56 (0.14, 2.24), *p* = 0.42	0.57 (0.21, 1.57), *p* = 0.28	1.01 (0.67, 1.52), *p* = 0.96	1.06 (0.29, 3.86), *p* = 0.93
**Luminex (ng/mg tissue)**				
Aβ_−1−42_	0.78 (0.52, 1.17), *p* = 0.23	0.86 (0.62, 1.19), *p* = 0.36	0.78 (0.52, 1.17), *p* = 0.23	1.13 (0.75, 1.71), *p* = 0.55
pTau	1.06 (0.78, 1.44), *p* = 0.72	1.02 (0.77, 1.35), *p* = 0.88	1.06 (0.78, 1.44), *p* = 0.72	1.10 (0.88, 1.39), *p* = 0.40
α-synuclein	1.09 (0.87, 1.36), *p* = 0.45	1.00 (0.78, 1.26), *p* = 0.97	1.09 (0.87, 1.36), *p* = 0.45	1.44 (0.85, 2.43), *p* = 0.17
**Isoprostanes (pg/mg tissue)**				
Isoprostanes^*^	1.04 (0.88, 1.22), *p* = 0.67	0.98 (0.76, 1.26), *p* = 0.85	N/A	N/A

* *Additional adjustment for duration of PMI*.

*All models are adjusted for age at death, sex, education, and the presence of any APOE ε4 alleles*.

We assessed Aβ signal (as a percentage of the tissue area occupied by the 6E10 antibody immunostain) in FFPE and frozen tissue samples from SMTG, IPL, hippocampus and PWM using immunohistochemistry, and measured levels of Aβ_1−42_ peptide in the samples of frozen tissue from the same four regions using a Luminex assay. TBI exposure was not associated with higher adjusted risk ratios for any of these measurements of Aβ compared to people without TBI (Table 6). The data is available at http://aging.brain-map.org/.

### α-Synuclein, Phospho-TDP-43, Iba1 and GFAP as Assessed by Immunohistochemistry and Luminex Assay in TBI_Froz_ Cohort

Levels of phospho-TDP-43, a pathological protein that has been implicated in CTE disease process and brain response to trauma (9) were measured in FFPE tissue in SMTG, IPL, hippocampus and PWM. We used immunohistochemistry and Luminex assay to measure levels of α-synuclein, a presynaptic protein present in Lewy bodies in frozen tissue samples in the same four regions. As shown in Table 6, TBI exposure was not associated with higher adjusted risk ratios for any of these measures compared to people without TBI.

To indirectly assess the extent of tissue reaction to injury and level of inflammation in the brains of people with a history of TBI and people without TBI history, we used immunohistochemistry to measure the levels of GFAP, a marker of astrocyte activation, and Iba1, a marker of activated microglia, in FFPE tissue samples from SMTG, IPL, hippocampus, and PWM. TBI exposure was not associated with higher adjusted risk ratios for any of these measures compared to people without TBI (Table 6). The data is available at http://aging.brain-map.org/

### Measures of Free-Radical Damage in TBI_Froz_ Cohort

Levels of free-radical damage in the cortical regions (SMTG and IPL) were assessed by measuring the concentrations of isoprostanes, products of free radical-catalyzed lipid peroxidation, in frozen tissue samples of SMTG and IPL. TBI exposure was not associated with higher adjusted risk ratios for isoprostane levels in any of these regions compared to people without TBI (see Table 6).

### Validation of pTau and Amyloid β_1−42_ Measurements

To confirm the validity of our measurements of pTau and Aβ_1−42_, we performed several additional data analyses. Because most study participants showed at least some level of AD pathological change (see Table 1), and AD was the most common neuropathological diagnosis in this cohort, we wanted to see if the regional patterns of pTau and Aβ_1−42_ deposition that we observed were consistent with AD pathology. As expected, higher levels of pTau as measured by Histelide were found in the STMG [median 0.11, interquartile range (IQR) 0.48–0.24] than in the MFG (median 0.08, IQR 0.03–0.15; *p* < 0.0001) or the IPL (median 0.06, IQR 0.02–0.12; *p* < 0.0001), controlling for sex, education, age at death, and the presence of 1 or more *APOE* ε4 alleles. Lower levels of Aβ_1−42_ were found in the IPL (median 0.23, IQR 0.08–0.46) than in STMG (median 0.28, IQR 0.11–0.52; *p* < 0.0001) or the MFG (median 0.27, IQR 0.09–0.52; *p* = 0.0004), controlling for sex, education, age at death, and the presence of 1 or more *APOE* ε4 alleles.

**Figure 5:**
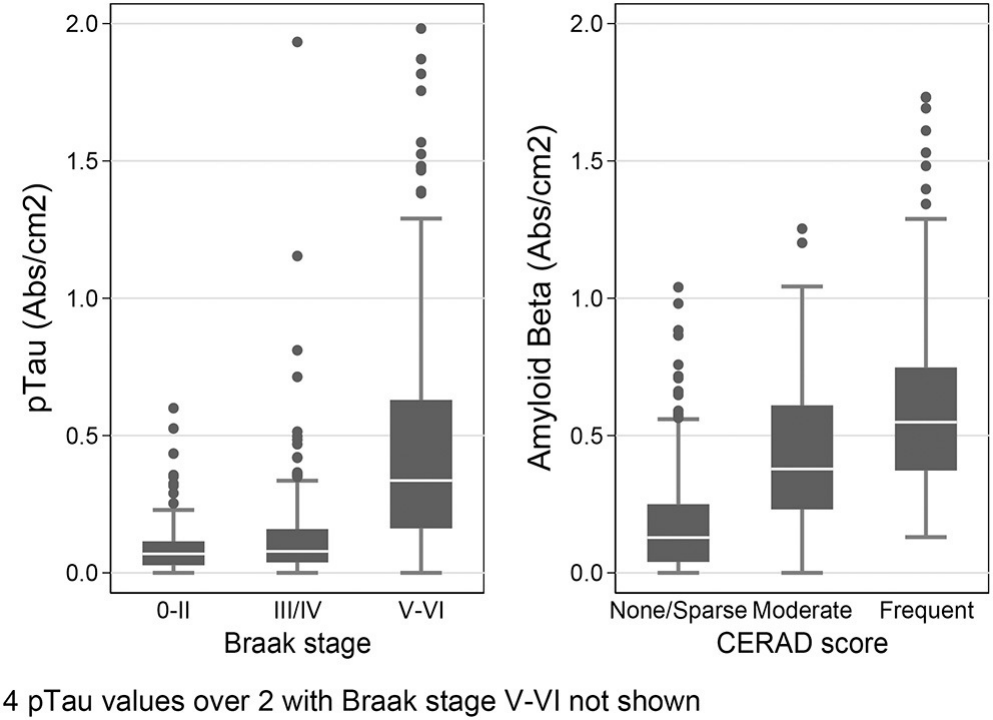
Histelide pTau levels stratified by Braak stage and Aβ_1−42_ levels (SI/cm^2^) by CERAD score in the SMTG in TBI_All_ cohort. In the box-and-whiskers plots, the box spans the 25th to 75th percentiles, with the median indicated in white. The whiskers define 1½ times the inter-quartile range; individual observations more extreme than this are indicated with dots.

To provide further validation of our results, we also stratified the data by the subjects' Braak stage, a staging system for AD based on the distribution of pTau-containing neurofibrillary tangles in the brain (62), and by CERAD score–another measure of AD pathology based on the density of Aβ-containing neuritic plaques in cerebral cortex (63). We hypothesized that in the temporal lobe samples (SMTG), final dementia status at the time of death and Braak stage should be associated with higher levels of both pTau and amyloid β_1−42_, and that the presence of 1 or more *APOE* ε4 alleles and CERAD score should be positively correlated with amyloid β_1−42_. As shown Figure 5, in the dataset obtained by Histelide, these relationships were all confirmed with our data (*p*-values 0.001–< 0.0001), and usually found in the MFG and IPL as well (Supplementary Table 2), adjusting for sex, education, age at death, and, except when it was the variable of interest, the presence of any *APOE* ε4 alleles.

**Figure 6:**
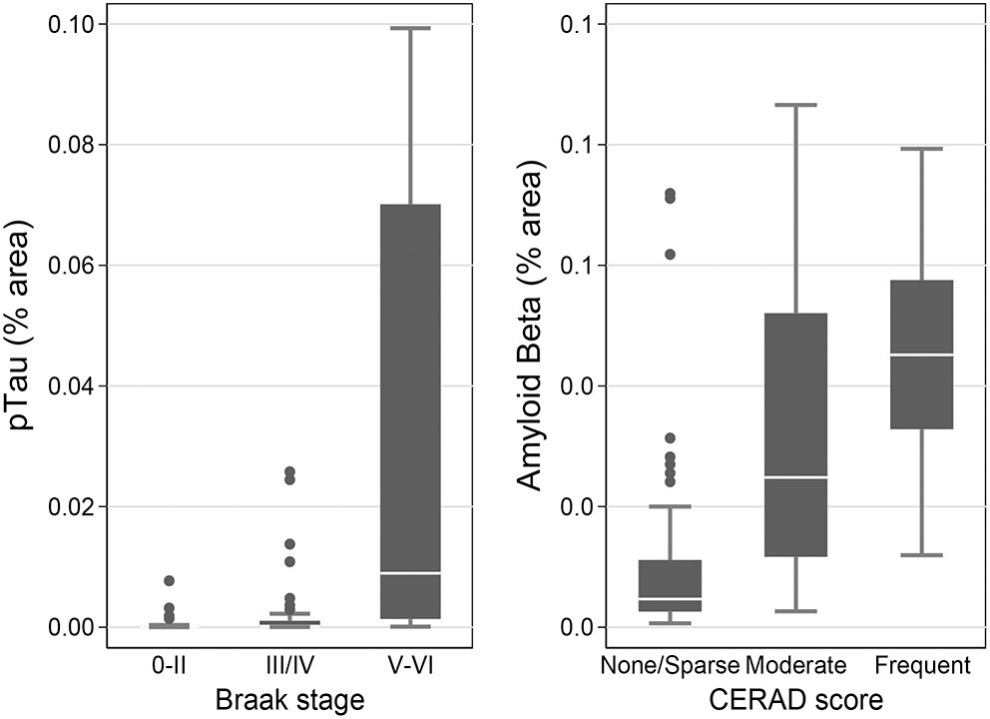
pTau levels stratified by Braak stage and Aβ levels by CERAD score as measured by IHC in the flash-frozen samples of STMG in TBI_Froz_ cohort. In the box-and-whiskers plots, the box spans the 25th to 75th percentiles, with the median indicated in white. The whiskers define 1½ times the inter-quartile range; individual observations more extreme than this are indicated with dots.

Measures of pTau obtained by immunohistochemistry and Luminex assay were all associated with Braak stage V/VI, which is indicative of high level of AD neuropathological change, in the SMTG (*p* < 0.0001 compared to stages 0/II; Supplementary Table 3 and Figure 6). The contrast for pTau (IHC, FFPE) between stage III/IV and 0/II was also statistically significant (*p* = 0.02). Measures of Aβ_1−42_ were all associated with CERAD scores in the SMTG (*p* ≤ 0.0003 for Moderate and *p* < 0.0001 for Frequent, compared to None; Supplementary Table 3 and Figure 6). Aβ (IHC, FFPE) and Aβ_1−42_ (Luminex) also distinguished CERAD score Sparse from None (*p* = 0.03 and *p* = 0.008, respectively). All models were adjusted for age at death, sex, education, and the presence of any *APOE* ε4 alleles.

### RNA- Sequencing in TBI_Froz_ Cohort

We did not find any genes with significant association to any measures of TBI in any of the regions based on several statistical tests. None of the statistical tests (see Methods) identified a single gene or component associated with TBI self-report, age at first TBI, longest loss of consciousness duration, or number of TBIs in any brain region tested, indicating that, using these methods, significant gene expression signatures of TBI are not identified in this data set (data not shown). The complete dataset is available at http://aging.brain-map.org/.

### CTE Pathology in the TBI_All_ Cohort

**Figure 7:**
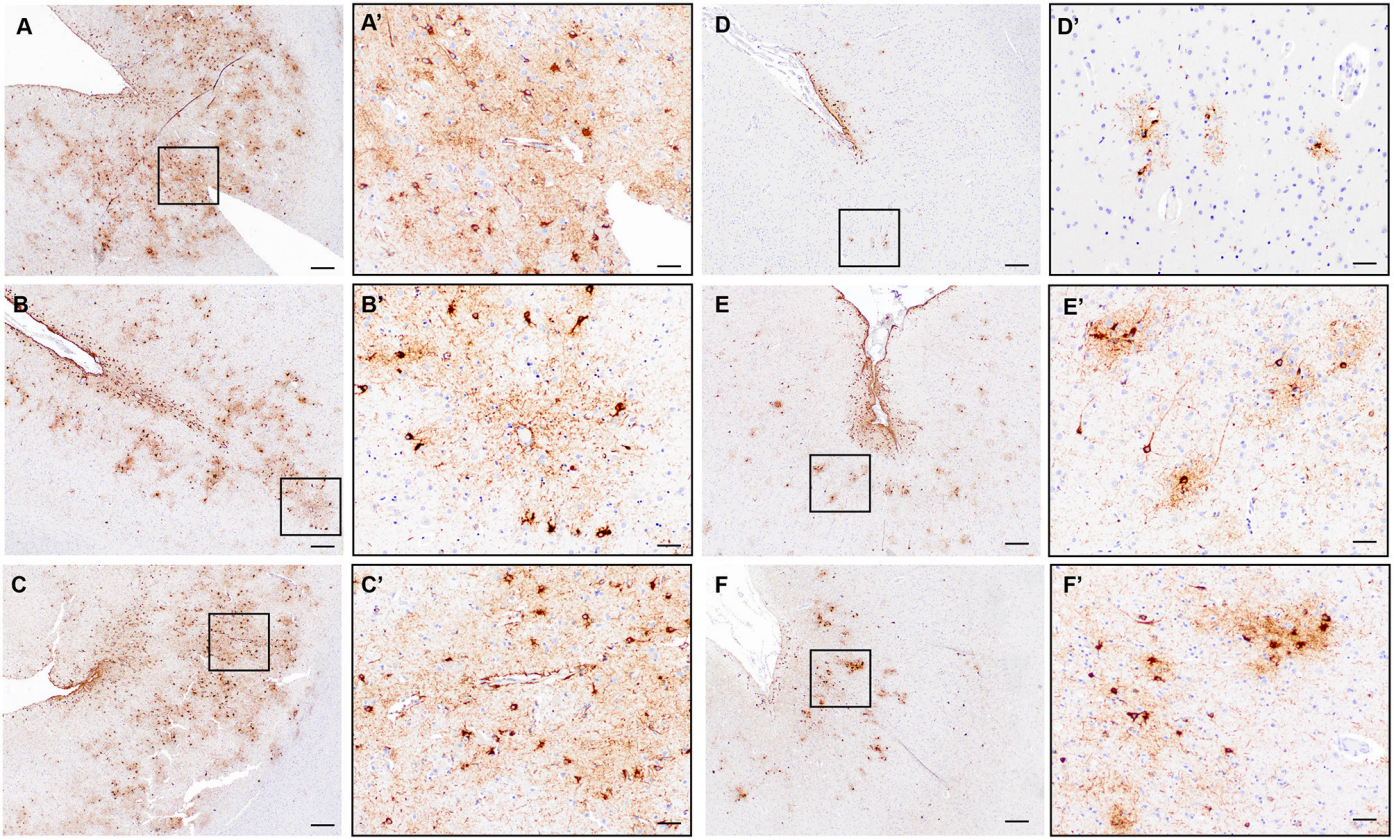
Evaluation of CTE neuropathologic change in the ACT cohort. **(A–C)** Representative images of the three cases with definite diagnostic features of CTE neuropathologic change characterized by clear perivascular pTau aggregates in neurons and glia at the depth of a sulcus and deep in the gray matter parenchyma. Black boxes indicate areas shown in **(A'–C')**. A/A'= Subject #2 in text, IPL; B/B'= Subject #1 in text, SMTG; C/C'=Subject #3 in text, IPL. **(D–F)** Representative images from cases without definite CTE but with irregular deep gray matter tau deposition. Distinct perivascular neuronal tau at the sulcal depth was not present to make a diagnosis of CTE. Black boxes indicate areas shown in **(D'–F')**. D = MFG; E = SMTG; F= IPL. Scale bars A-F = 200 μm; A'-F' = 50 μm.

To assess the prevalence of CTE pathology in our autopsy cohort, we used the Histelide slides immunolabeled with an antibody to pTau (AT8) to screen each section for increased density of sulcal pTau over other areas of the cortical section. Concerning regions with deposition of sulcal pTau in the deep gray matter were additionally evaluated with standard AT8 immunohistochemistry. Only three cases out of 532 (0.6%) demonstrated the neuropathologic features of CTE (Figures 7A–C). In all cases, CTE changes were only observed in a single region in temporal or parietal lobe (SMTG for one case and IPL for two cases).

Subject #1 was male, 82 years old and not demented at the time of death. Obituary was unavailable. Neuropathological diagnosis was AD. Subject #2 was male, 100 years old and demented at the time of death. Obituary was available, and there was no indication of either contact sport or military history in the obituary. The subject had depressive episodes later in life, was cognitively impaired in the last 7 years of life and sustained several falls during that time. Neuropathological diagnosis was vascular brain injury. Subject #3 was male, 94 years old and demented at the time of death. Obituary review indicated military service, but no contact sport exposure. The subject was treated for clinical late in life depression, was cognitively impaired in the last decade of life and sustained multiple falls during that time. Behavioral disturbances during the last 7 years of life were consistent with the clinical diagnosis of AD, which was later confirmed by neuropathological evaluation.

None of the three participants with CTE neuropathologic change had a history of previous TBI with LOC before developing dementia in the 8th or 9th decade of life, and none of the subjects were specifically evaluated for chronic traumatic encephalopathy syndrome (TES). Behavioral disturbances noted in subjects #2 and #3 were consistent with vascular/mixed dementia in case #2 and Alzheimer's type dementia in case #3. All three subjects were carriers of one copy of *APOE* ε4 allele.

Twenty-four additional participants showed pTau deposits at the depth of the sulcus, most often superficial subpial thorn-shaped astrocytes, in one or more cortical sections. Twenty of the 24 subjects were male, seven of them had a history of TBI. The average age at death was 86.9 years; nine were demented. Nine were diagnosed with AD, two with LBD and nine with vascular brain injury. Obituaries were available for 11 participants. According to the obituary data, four were military veterans and one played a contact sport. Three of these cases had additional deep gray matter tau deposition, but a convincing neuronal and perivascular distribution was not identified (Figures 7D–F), and therefore these cases were not considered to have definite CTE. Many of the remaining cases, as expected, had significant pTau pathology in a diffuse pattern in association with neuritic plaques consistent with AD neuropathologic change and lacked preferential accumulation of pTau in depth of sulcus.

## Discussion

This study was designed to assess the delayed consequences of TBI with LOC in a community-based sample. We not only evaluated the cases for CTE neuropathologic change but performed a comprehensive multimodal assessment to examine for significant alterations in multiple pathologic proteins, inflammation and gene expression later in life.

We used the Histelide method to measure levels of pTau and Aβ_1−42_ in samples of frontal, temporal, and parietal cortex, and did not see a significant effect of history of prior TBI on levels of either protein. One caveat in assessing pTau and Aβ_1−42_ in the ACT autopsy cohort is that these are hallmark pathologic peptides of AD, which is highly prevalent due to the advanced age of most ACT decedents (64, 65). This confounding variable could have introduced variability to the data to obscure small differences related to TBI between groups. To address this potential impact, we designed and carried out additional experiments and analyses in a cohort of ACT participants with TBI w/LOC and non-exposed matched controls. Because there is a potential effect of previous neurotrauma on the development of AD (21, 66, 67) and other neurodegenerative diseases (26, 27, 68, 69), we chose not to match our subjects on markers of AD neuropathologic change or other neurodegenerative disease pathologies. Instead, we matched them on age and sex, factors that are known to affect the prevalence of AD (70–73) and can influence the rate of accumulation of pTau and Aβ in the brain (74, 75). However, we still did not detect any significant TBI w/LOC-related differences in pTau and Aβ_1−42_ levels, as assessed by Histelide, across the whole TBI-exposed ACT cohort and in a smaller subset of ACT participants with the most significant TBI exposures (TBI w/LOC>1 h and/or multiple TBI w/LOC). In another matched cohort of people with short PMI who had frozen tissue available for analyses, we also did not detect any differences in Aβ species between those with TBI w/LOC and the unexposed groups, but we did observe significantly higher levels of hippocampal, but not cortical, pTau in the TBI group in 2 out of 3 different types of assays. Given the total number of comparisons that were carried out, this result should be taken with caution. However, it may be in line with the increased tau pathology that has been reported in studies of more severe TBI at chronic time points after injury.

Our primary analyses relied on Histelide measures, a novel highly sensitive method of quantifying immunoreactivity in FFPE tissue. We conducted a series of sensitivity analyses to illustrate that Histelide is indeed quantifying the underlying distribution of pathology upon which the traditional gold-standard indices of neurodegenerative disease pathology are based. Each of our measurements of pTau and Aβ species were validated by analyzing subjects by AD neuropathologic change (63, 76), where we consistently observed a significant positive correlation between levels of pTau and Aβ and Braak stage and CERAD score. We detected elevated levels of both pathological proteins in subjects with dementia, as well as in carriers of *APOE* ε4 allele, trends that have been described previously (77, 78). Taken together, these observations confirm that the methods we used here were adequate to detect differences in the levels of pTau and Aβ in this cohort.

Chronic repetitive neurotrauma has been associated with increased pTau accumulation in the brain, at least in some cases (79–82), and pTau deposits are considered a major diagnostic feature of CTE (7, 8). However, much less is known about the effect of a single or a few mild concussive events. While a study by Uryu et al. reported increased pTau immunoreactivity in a small percentage of cases with recent TBI (83), Smith et al. did not find an increase in number of NFTs in acute fatal cases of a single TBI compared to age-matched controls (84). While post-concussive pTau accumulation has been previously demonstrated in some animal studies (82, 85, 86), other reports did not support this observation (87–89). In this study, history of remote TBI w/LOC does not appear to have a consistent, significant effect on cortical levels of pTau using quantitative approaches, but small effects of pTau related to TBI exposure could be masked by significant AD-neuropathological burden in this cohort.

We did not observe a relationship between the history of TBI with LOC and Aβ levels in the current study. APP, a precursor of amyloid β, is known to be involved in the acute response to brain injury (83), and several studies have reported increased accumulation of Aβ shortly after TBI in humans (38, 83, 90), although this phenomenon appears to occur only in a subset of cases with TBI exposure (81). A previous study by our group carried out in ACT, one of the largest studies on the subject, which included the majority of cases analyzed in the current report did not find an association between remote TBI with Aβ load as reflected by CERAD score (27). While Aβ deposits are common in severe CTE, they are often absent in milder cases (91, 92). Because none of the cases in this study had a known history of severe repetitive TBI or neuropathological changes suggestive of high stage CTE, it is not surprising that we did not observe a significant association between the history of TBI and cerebral Aβ.

We did not limit assessments to Aβ and pTau, but also measured levels of other pathologic peptides, such as phosphorylated α-synuclein and phospho-TDP-43, assessed markers of the inflammatory response (GFAP and Iba1), evaluated for oxidative stress (isoprostanes) and performed RNA-sequencing in an attempt to cast a wide net to detect TBI-related neuropathological changes in the ACT autopsy cohort. We did not detect any differences between groups defined by exposure to TBI w/LOC by any of the measurements in a smaller matched cohort with high quality frozen tissue samples (49 subject pairs), which might have provided insufficient statistical power to detect small differences.

All Histelide slides immunolabeled with pTau antibody were screened for neuropathological signs of CTE. For the whole cohort, MFG, SMTG, and IPL were screened, as standard samples taken for the entire cohort for recommended AD neuropathologic change workup. In a subset of cases with TBI w/LOC >1 h and/or multiple TBI w/LOC, additional regions, including some form the contralateral hemisphere, were also examined. However, superior frontal gyrus and temporal pole, sites recommended for CTE screening by NINDS/NIBIB consensus meeting criteria (9) are not routinely sampled and could not be assessed for this large number of cases. While we evaluated a minimum of three cortical regions in one hemisphere, recommended sampling protocols would have expanded that to five cortical regions bilaterally, or ~3 times more samples than our minimum per case. The three regions that we examined are known to be disproportionately affected by head trauma (8), and a very small subset of cases in this study (0.6%) displayed the neuropathologic changes that define CTE (each identified in a single brain region in all cases). The lack of a more extensive evaluation of additional regions of the brain may have contributed to the relatively low CTE prevalence in the current study, compared to earlier reports in other population-based studies: 11.9% in the study by Ling et al. (93), and 35% in the study by Noy et al. (94). However, even if we found three times more cases in the recommended 10 sampling areas, we would still be below 2% of the ACT cohort, and a more recent study, using diagnostic criteria similar to ours, reported no CTE cases in a community-based sample of 320 (95). Even if sensitivity to detect CTE was increased in a region not sampled here, it seems unlikely, based on the described distribution patterns of lesions of CTE, that the prevalence in the ACT cohort would approach levels seen in selected cohorts.

For the diagnosis of CTE neuropathology, cases of depth of sulcus pTau were not classified as CTE if the pTau was strictly astrocytic, was superficial/subpial only, or was not perivascular, all of which can be attributed to age-related tau astrogliopathy (ARTAG) (96, 97). Thus, of the 27 cases with depth of sulcus pTau lesions, 24 had features not diagnostic for CTE and more suggestive of ARTAG, a highly prevalent tauopathy of aging. None of the three CTE cases had a history of TBI w/LOC. Two of the three participants sustained multiple falls after developing dementia in in the 8th and 9th decade of life, but had no record of LOC. Observations from several large-scale neuropathology studies support the notion that diagnostic features of CTE can be present in cases without prior history of known head trauma with LOC. In the 2016 study by Noy and colleagues, more than one-third of cases displaying neuropathological signs of CTE with only focal sulcal deposition, similar to our cases, did not have an explicit history of head trauma (94). Similarly, in earlier reports by Ling et al. (93), Koga et al. (96), and Bieniek et al. (12) not every case displaying neuropathological signs of CTE had a documented history of head trauma. It is important to note that none of the cases in the current study had the level of pathologic changes suggestive of high stage CTE, but future consensus meetings are necessary to further refine diagnostic and staging criteria for CTE.

Several studies have described an association between neurotrauma and dementia in younger individuals. Nordstrom and colleagues (18) found a link between the history of TBI and the onset of dementia before the age of 65, while other authors reported increased risk of dementia and lower age of onset of cognitive impairment in subjects with moderate to severe TBI (13, 20). Because the enrollment in the ACT study is limited to individuals who do not have dementia at the age of 65, the ACT study is likely inadvertently selected against subjects who sustained a severe TBI or were exposed to repetitive head injury, and developed CTE and traumatic encephalopathy syndrome (TES) as a result. According to a previous report, CTE-associated cognitive symptoms could appear as early as in the 3rd decade of life, and over half of the subjects in the cognitive-predominant group in that study were demented at the time of death, which on average occurred at 69 years of age (98). The relatively short life span of subjects with symptomatic CTE [57 years in the study of Stern et al. (98)] creates significant difficulties in interpreting CTE findings in any cohort established to study brain aging. However, because of the high age and cognition cutoff of the ACT study, even people who sustained a single mild to moderated TBI, but had a higher than average susceptibility to the detrimental effect of neurotrauma on cognition, could have been excluded from the ACT. At present time, it is difficult to estimate the effect of this potential bias on our results. The issue is complicated by the fact that a number of studies reported no association between a history of TBI and development of AD, the most common type of dementia (33, 99, 100), and no association between TBI and dementia was reported earlier in the ACT cohort (33) and other smaller cohorts (101, 102). Another study in the ACT cohort, which included most of the cases analyzed here, did not find an association between the history of TBI and incidence of dementia or AD. The authors did report an association between TBI and incidence of PD, presence of LBD and microvascular injury (27), but these pathologies were less likely to affect the enrollment in the study unless accompanied by dementia. Another potential bias of the ACT study that warrants consideration is that all ACT study participants were members of an HMO and are therefore not a true representation of the general population. One can speculate that individuals in whom previous TBI significantly impacted general health and longevity were less likely to be recruited into ACT, creating a study bias toward participants, who were, on average, less susceptible to the pathological consequences of brain injury. This would be particularly true for individuals who sustained a severe and/or repeated TBI and developed TES, because as reported in an earlier study by Stern et al. participants with behavioral/mood variant of TES died at the average age of 51, and many of them developed symptoms decades earlier (98).

The assessment for TBI exposure history in ACT participants was designed at its inception three decades ago and does not perfectly conform to current concepts of TBI severity, which may affect direct comparisons to other studies. Although LOC duration of 30 min is commonly used to differentiate a mild from a moderate TBI, this time point was not available in the ACT study. Therefore, we chose LOC of >1 h to define the high TBI exposure group. Further, TBI ascertainment methods used in ACT prevented us from thoroughly characterizing subconcussive exposure and additional information (i.e., obituary) regarding military and contact sport participation was limited (although was not associated with any variables in this study in the participants for whom some exposure data was identified—data not shown). This problem is not unique to these types of assessments in cohorts not selected for TBI exposure and can lead to misclassification of participants with history of mild TBI without LOC and/or cases with repetitive subconcussive exposure being erroneously placed in the TBI unexposed group. Other studies before us faced the same issues. In their 2020 report, Bieniek et al. examined 750 autopsy cases from the Mayo Clinic Tissue Registry and relied on obituaries and high school yearbooks to assess sport participation (12). The authors found diagnostic CTE lesions in 1.3% of cases with no documented history of sports participation and in 5% of former athletes. We cannot directly compare our finding to the results of Bieniek et al. because of the uncertainty of sports exposure in the ACT study, where a little over half of the cases had an obituary available, while a presence of an obituary and/or yearbook was an inclusion criteria in the former study. Nevertheless, the prevalence of CTE in the ACT cohort as assessed by us appears lower than even in the unexposed group in the report of Bieniek and colleagues. A possible explanation for this discrepancy may be the relatively strict inclusion criteria of the ACT study, as described above. Average age at death in the study of Bieniek et al. was 68 and 64 y. o. for athletes and non-athletes, respectively, while the ACT study only accepted individuals that were at least 65 y. o. and non-demented at the time. Another potential factor could be the slight differences in the cortical regions examined (frontal and temporal, bilateral for at least one region in the study by Bieniek et al. vs. frontal, temporal and parietal in the current study).

Ongoing and future prospective cohort studies that include detailed methods to assess TBI exposure history will be essential to understand the degree to which false negative cases confound data analyses. Finally, the types of TBI exposures, particularly in sports and the military, have changed over the time period representing the lifespan of most of the ACT participants, and safety protocols and equipment have evolved as well. The relevant exposures studied in ACT need to be compared with those of subsequent generations to understand how changing exposure impacts future risk for neuropathological and functional outcomes.

Despite the aforementioned limitations, results from this community-based study allow us to draw several conclusions which should be applicable to the general population. We conclude that, on average, a remote single or rare TBI w/LOC is not associated with significant neurodegenerative disease-related changes in the brain in late life. Better characterization of TBI exposures, increasing numbers of cases and assay sensitivities, and extending sampling strategies to ensure inclusion of at risk brain regions, will be important to continue to explore relative risk for TBI-related late neuropathological changes. We also conclude that neuropathological features of CTE, which have been historically associated with the history of chronic repetitive brain trauma, are not highly prevalent in a community-based cohort of brain aging and neurodegeneration. Overall, while it appears that remote TBI w/LOC does not seem to leave a dramatic neuropathological signature on brain structure or its molecular composition, this finding raises more questions than it answers. The absence of clear general trends highlights the importance of uncovering differences in individual susceptibility to the effects of neurotrauma, with the goal of eventually developing targeted intervention and treatment strategies.

## Data Availability Statement

The original contributions presented in the study are publicly available. This data can be found at NIAGADS: https://www.niagads.org/datasets/ng00059.

## Ethics Statement

The studies involving human participants were reviewed and approved by University of Washington Insitutional Review Board. The patients/participants provided their written informed consent to participate in this study.

## Author Contributions

CK, EL, and KD-O'C: study concept and design. SR, NC, LH, KR, AW, EC, XL, AB, JM, DM, KD-O'C, and CK: data acquisition. LG, SR, NP, JM, and EM: data analysis. CK, AN, and SR: neuropathological assessment. All authors: data interpretation and drafting of manuscript.

## Conflict of Interest

The authors declare that the research was conducted in the absence of any commercial or financial relationships that could be construed as a potential conflict of interest.
