# Supplementary material for: The Delayed Neuropathological Consequences of Traumatic Brain Injury in a Community-Based Sample
Source: Front Neurol. 2021 Mar 16;12:624696. doi: 10.3389/fneur.2021.624696 (PMC8008107; doi:10.3389/fneur.2021.624696)
Supplement: Supplementary file 1 [file Table_1.DOCX]

Supplementary Table 1. Relative Risks with 95% confidence intervals for TBI with LOC greater than an hour or multiple TBI (n=27), compared with no TBI (n=54). Levels of pTau were assessed by histelide. All models are adjusted for age at death, sex, education, and the presence of any *APOE* ε4 alleles.

| Region | Relative Risk  (95% CI) | p-value |
| --- | --- | --- |
| Occipital Cortex | 1.05 (0.68, 1.61) | 0.83 |
| Cingulate cortex | 1.04 (0.64, 1.70) | 0.87 |
| Hippocampus | 1.17 (0.79, 1.73) | 0.42 |
