# Supplementary material for: The Delayed Neuropathological Consequences of Traumatic Brain Injury in a Community-Based Sample
Source: Front Neurol. 2021 Mar 16;12:624696. doi: 10.3389/fneur.2021.624696 (PMC8008107; doi:10.3389/fneur.2021.624696)
Supplement: Supplementary file 2 [file Table_2.DOCX]

Supplementary Table 2. Relative Risks with 95% confidence intervals for variables hypothesized to be associated with pTau and amyloid β_1-42_ accumulation in the SMTG, as assessed by histelide. All models were adjusted for age at death, sex, education, and except when it was the variable of interest, the presence of any *APOE* ε4 alleles.

| Validation variable | Analyte/ | Relative Risk  (95% CI) | p-value | Relative Risk  (95% CI) | p-value | Relative Risk  (95% CI) | p-value | Other Regions* |
| --- | --- | --- | --- | --- | --- | --- | --- | --- |
| Dementia |  | Any Dementia |  |  |  |  |  |  |
|  | pTau | 4.16 (2.91, 5.97) | < 0.0001 |  |  |  |  | IPL |
|  | Amyloid β_1-42_ | 1.48 (1.10, 1.98) | 0.0096 |  |  |  |  |  |
|  |  |  |  |  |  |  |  |  |
| APOE ε4 |  | Any ε4 alleles |  |  |  |  |  |  |
|  | Amyloid β_1-42_ | 1.67 (1.44, 1.93) | < 0.0001 |  |  |  |  | MFG, IPL |
|  |  |  |  |  |  |  |  |  |
| Braak |  | III/IV |  | V/VI |  |  |  |  |
|  | pTau | 1.75 (1.31, 2.34) | 0.0002 | 7.02 (4.94, 9.98) | < 0.0001 |  |  | MFG, IPL |
|  | Amyloid β_1-42_ | 1.41 (1.15, 1.74) | 0.0010 | 2.39 (1.94, 2.96) | < 0.0001 |  |  | MFG, IPL |
|  |  |  |  |  |  |  |  |  |
| CERAD |  | Sparse |  | Moderate |  | Frequent |  |  |
|  | Amyloid β_1-42_ | 2.74 (2.15, 3.48) | < 0.0001 | 4.56 (3.62, 5.75) | < 0.0001 | 6.46 (5.13, 8.14) | < 0.0001 | MFG, IPL |

* Statistically significant associations in MFG or IPL.
