# Supplementary material for: The Delayed Neuropathological Consequences of Traumatic Brain Injury in a Community-Based Sample
Source: Front Neurol. 2021 Mar 16;12:624696. doi: 10.3389/fneur.2021.624696 (PMC8008107; doi:10.3389/fneur.2021.624696)
Supplement: Supplementary file 3 [file Table_3.DOCX]

Supplementary Table 3. Relative Risks with 95% confidence intervals for the relationship between Braak stage and pTau or between the CERAD score and amyloid β_1-42_ in the SMTG, as assessed by histelide. All models were adjusted for age at death, sex, education, and the presence of any *APOE* ε4 alleles.

| Validation variable | Analyte, Method | Relative Risk  (95% CI) | p-value | Relative Risk  (95% CI) | p-value | Relative Risk  (95% CI) | p-value |
| --- | --- | --- | --- | --- | --- | --- | --- |
| Braak |  | III/IV |  | V/VI |  |  |  |
|  | pTau, IHC-FFPE (% area) | 1.93 (1.11, 3.35) | 0.0191 | 5.89 (3.59, 9.68) | < 0.0001 |  |  |
|  | Tau-2, IHC-FFPE (% area) | 1.03 (0.67, 1.59) | 0.8767 | 2.94 (1.89, 4.57) | < 0.0001 |  |  |
|  | pTau, Luminex (ng/mg tissue) | 0.97 (0.68, 1.39) | 0.8817 | 2.35 (1.58, 3.48) | < 0.0001 |  |  |
|  |  |  |  |  |  |  |  |
| CERAD |  | Sparse |  | Moderate |  | Frequent |  |
|  | Aβ, IHC-FFPE (% area) | 1.57 (1.05, 2.35) | 0.0264 | 2.51 (1.66, 3.80) | < 0.0001 | 3.41 (2.34, 4.96) | < 0.0001 |
|  | Aβ, IHC-Flash-frozen (% area) | 1.58 (0.68, 3.69) | 0.2885 | 3.65 (1.80, 7.39) | 0.0003 | 4.69 (2.43, 9.06) | < 0.0001 |
|  | Aβ1-42, Luminex (pg/mg tissue) | 5.05 (1.52, 16.77) | 0.0083 | 9.32 (3.12, 27.81) | 0.0001 | 10.05 (3.30, 30.60) | < 0.0001 |
